# Whole-Genome Shotgun Sequencing and Annotation of *Mycobacterium tuberculosis* MTB221/11 Isolated from a Cerebrospinal Fluid Sample in Malaysia

**DOI:** 10.1128/genomeA.00376-16

**Published:** 2016-06-30

**Authors:** Rahizan Issa, Valentinus H. Seradja, Mohd Khairul Hafizi Abdullah

**Affiliations:** Bacteriology Unit, Infectious Disease Research Centre, Institute for Medical Research, Jalan Pahang, Kuala Lumpur, Malaysia

## Abstract

Here, we report of the annotated genome sequence of *Mycobacterium tuberculosis* MTB221/11. The organism was isolated from the cerebrospinal fluid of a patient in Malaysia.

## GENOME ANNOUNCEMENT

In Malaysia, tuberculosis (TB) is a disease of major public health significance. In 2014, the World Health Organization reported an estimated incidence of 80 new cases annually per 100,000 population (1). According to a survey done at chest clinics, 10 to 11% of TB cases were classified as extra pulmonary tuberculosis (2). Here, we report the annotated whole-genome shotgun sequence of *Mycobacterium tuberculosis* 221/11, isolated from a sample of cerebrospinal fluid from a relapse TB male patient. 

The total genomic DNA of *M. tuberculosis* 221/11 was extracted using QIAxtractor and reagent packs (Qiagen, Düsseldorf, Germany) according to the manufacturer’s instructions. DNA concentrations were measured using a NanoVue Plus spectrophotometer (GE Health Care, Buckinghamshire, United Kingdom). 

The Illumina MiSeq platform was used for the whole-genome shotgun sequencing, which resulted in 6,320,131 filtered reads with an average read length of 101.7 bp and approximately 146-fold coverage. The filtered reads were *de novo* assembled with CLC Genome Workbench version 6.0.1, generating 161 contigs (*N*_50_, 72,213). The genome contains a total of 4,362,451 bp with a G+C content of 65.59%.

The NCBI Prokaryotic Genome Annotation Pipeline was used for annotation. The genome was identified has having 4,152 genes, 3,964 coding sequences, and 3,964 pseudogenes. Three types of rRNA (5S, 16S, and 23S) were annotated, and one rRNA of each type was present. One CRISPR array was annotated in this strain.

The SpolPred software was used to determine the spoligotype of *M. tuberculosis* 221/11. It was determined as 777777777760731, which corresponds to Euro-American lineage.

### Nucleotide sequence accession number.

This whole-genome shotgun project has been deposited in DDBJ/ENA/GenBank under the accession number LFLI00000000. The version described in this paper is the first version.
